# Teacher-reported kindergarten development among children exposed to prescription opioids during pregnancy

**DOI:** 10.1371/journal.pone.0355101

**Published:** 2026-08-06

**Authors:** Omaymah Abulannaz, Chelsea A. Ruth, Andi Camden, Marni Brownell, M. Florencia Ricci, Roxana Dragan, Depeng Jiang, Geert ‘t Jong, Lauren E. Kelly

**Affiliations:** 1 Department of Pharmacology and Therapeutics, University of Manitoba, Winnipeg, Canada; 2 Department of Pharmacology, Jordan University of Science and Technology, Irbid, Jordan; 3 Department of Pediatrics and Child Health, University of Manitoba, Winnipeg, Canada; 4 Manitoba Centre for Health Policy, University of Manitoba, Winnipeg, Manitoba, Canada; 5 Department of Health and Society, University of Toronto Scarborough, Toronto, Canada; 6 ICES, Toronto, Canada; 7 Department of Community Health Sciences, University of Manitoba, Winnipeg, Canada; 8 Children’s Hospital Research Institute of Manitoba, Winnipeg, Canada; Karolinska Institutet, SWEDEN

## Abstract

**Background:**

Few studies have provided inconsistent findings on the neurodevelopmental impact of prescription prenatal opioid exposure (PPOE). In this study, we examined the association between PPOE and developmental vulnerability later in childhood.

**Methods:**

This was a matched cohort study using the Manitoba Population Research Data Repository. This cohort included dyads with children born in Manitoba, Canada between 2005–2011 with follow up until 2016. Each exposed child was matched to up to four controls based on birthing parent’s age, gestational age, sex, kindergarten year, and income quintile. A sibling cohort was created to compare the outcomes between exposed and unexposed siblings. PPOE was defined as filling ≥ 2 prescriptions for opioids at any time during pregnancy. Of the 973 children with PPOE, the majority (94%) had low-potency opioid exposure, primarily codeine. The Early Development Instrument (EDI) was used to assess developmental vulnerability in kindergarten. Multivariable logistic regression models were used to adjust for confounders.

**Results:**

Of the 103,905 eligible children, 51% of the exposed group (n = 973) were developmentally vulnerable in 1 or more domains, compared with 32% of the control group (n = 3,814) (Adjusted Odds Ratio [AOR] 1.46; 95% CI 1.23-1.71). Children in the exposed group had a significantly higher likelihood of being vulnerable in all domains: physical health and well-being (AOR 1.31; 95%CI 1.08-1.59), communication skills and general knowledge (AOR 1.53;95%CI 1.25-1.88), language and cognitive development (AOR 1.53;95%CI 1.25-1.86), social competence (AOR 1.60;95%CI 1.32-1.95); emotional maturity domain was the least affected (AOR 1.25;95%CI 1.01-1.55). There was a higher risk of developmental vulnerability in children exposed to opioids in early pregnancy, longer durations and higher opioid dose. In the sibling cohort, no significant differences were observed in the EDI outcomes between exposed and unexposed siblings (AOR 0.92; 95% CI 0.58-1.46).

**Conclusion:**

In the matched cohort study, exposure to prescription opioids during pregnancy was associated with a higher risk of developmental vulnerability later in childhood. In the sibling analysis, no differences were observed. These data strongly suggest that families should be offered early and long-term developmental screening and family-centered support.

## Introduction

Opioid use has increased dramatically and presents a major public health concern in North America [1]. The majority of women misusing opioids are within childbearing age [2]. In Manitoba, administrative health data identified opioid prescriptions in 7.7% of pregnancies in 2013 [3]. The outcomes of neonates following prescription prenatal opioid exposure (PPOE) are well-recognized in the literature and include increased risk for prematurity, low birth weight and neonatal opioid withdrawal syndrome (NOWS) [4]. However, a recent systematic review has identified gaps in evidence evaluating the long-term neurodevelopmental outcomes following PPOE [5]. These include inadequate control over confounding factors such as environmental, social and genetic variables that can impact the long-term neurodevelopmental outcomes of PPOE, small sample sizes, heterogeneity among included studies emerging from variations in type of opioid exposure, study populations, and assessment tools hindering meta-analysis.

Few studies have provided inconsistent insights into the potential neurodevelopmental effects of PPOE. Studies yielded conflicting results regarding Bayley Scales of Infant and Toddler Development scores [5–9]. Developmental vulnerability scores are important indicators of children’s neurodevelopment and can signal areas for intervention with the potential to improve long-term outcomes [10]. A few recent studies evaluated the educational outcomes among children with NOWS and reported that neonatal withdrawal was strongly associated with poor and deteriorating school performance, and significantly increased the likelihood of developing a subsequent educational disability [11, 12]. Studies conducted in the United States and Australia assessed educational outcomes in children with NOWS; however, because not all infants with PPOE meet the diagnostic criteria for NOWS, the expected outcomes following PPOE are not well understood.

We hypothesize that children with PPOE are at a higher risk of having developmental vulnerability than children unexposed to prescription opioids during pregnancy. To answer this question, we conducted a retrospective matched cohort study to investigate the association between PPOE and neurodevelopmental outcomes at 6 years of age. Furthermore, we evaluated the effect of PPOE on developmental outcomes among siblings where one or more of the siblings were exposed and one or more were unexposed within the same family. By conducting a sibling analysis, we aimed to mitigate the influence of unmeasured confounding factors stemming from the complex interplay of biological, social, and environmental elements affecting children exposed to opioids in utero.

## Methods

### Design and subjects

This is a retrospective matched cohort study using the Population Research Data Repository at the Manitoba Center for Health Policy (MCHP), University of Manitoba, Canada. Data are de-identified, population-wide and linkable across the health, social, and educational sectors, allowing for robust pharmacoepidemiological research [13–15]. This study is STROBE-compliant, and ethics, privacy and access approvals were obtained from the University of Manitoba Health Research Ethics Board (H-REB). The ethics committee approved a waiver of informed consent. Because the data were fully anonymized before analysis. Data access was obtained on March 10, 2023.

The databases used in this study were the Hospital Discharge Abstract Database; Drug Program Information Network; Medical Services; Manitoba Health Insurance Registry; Early Development Instrument (EDI); Families First Screen; Child and Family Services; and Shared Health Diagnostic Services. Linkage across the datasets was completed using a scrambled personal health identification number. The repository data have been widely and extensively used for population health research and their validity of dyad linkages has been well-documented [16–19].

We included birthing parent-child dyads with live hospital births between January 1, 2005 and December 31, 2011, in Manitoba. Dyads were excluded for the following factors: birthing parent with no health coverage 9 months before birthdate, twin births, missing gestational age, or<34 weeks, birthing parent age < 14, missing income quintile data, birthing parent who had only one opioid prescription, those with NOWS diagnosis and no opioid prescriptions, children with no recorded EDI, and dyads that could not be matched.

### Exposure

The exposure group includes birthing parents who have filled at least two outpatient prescriptions for an opioid any time during pregnancy. Opioid analgesics and Opioid Agonist Therapies (OAT) for the treatment of opioid use disorder were included. Each child in the exposure group was matched to up to four controls based on birthing parent’s age, gestational age, sex, kindergarten year, and income quintiles. Gestational age matching was categorized as moderate to late preterm (35–36 weeks), term (37–41 weeks) and post-term (42 + weeks). Birthing parent age was categorized as 14–19,20–24,25–29,30–34,35–39, and 40 + years. Out of the total cases, 927 were successfully matched with 1–4 controls, 22 cases were matched with 3 controls, 16 cases with 2 controls, and 8 cases with 1 control. Cumulative opioid dose was measured using morphine milligram equivalents (MME) to allow for meaningful comparison across different opioid medications. We converted the total opioid amount to MME using the morphine equivalent ratios [3]. For cumulative MME analysis, methadone was excluded because most methadone use was from compounded products for which MME cannot be determined.

### Outcomes

The Early Developmental Instrument (EDI) was used to assess developmental health of the cohort. EDI is a well-validated 103-component kindergarten teacher-completed questionnaire, that encompasses five developmental domains: physical health and well-being, language and cognitive development, emotional maturity, communication skills and general knowledge, and social competence [20]. Kindergarten teachers complete EDI questionnaires for students enrolled in the public school system during the latter half of the year. EDI was first administrated in 2005 and data are available every second school year between the years 2006–2017. EDI has been used globally as a population-based indicator of children’s developmental health and has a high psychometric property [20–22]. EDI domain scores are represented as a numerical value ranging from 0-10, alongside a percentile ranking based on the Canadian population norms. If a child’s score ≤ the 10^th^ percentile of the Canadian population norms for any one or more domains, they are classified as being developmentally vulnerable.

For the sibling cohort, we compared children with PPOE with their unexposed siblings living in the same home, and in an attempt to match the environments we excluded children who were taken ever into care. Birthing parents’ encrypted Personal Health Identification numbers were used to specify siblings.

### Covariates

In the main analysis the models adjusted for birthing parent mental disorders, birthing parent diabetes, gestational age, sex and children’s receipt of child protective services. Data on prenatal alcohol use, substance use, and smoking were obtained from the Families First Screen (FFS) database. A large proportion of the cohort had missing data for these variables (50%); accordingly, they were not included in the main analysis. We explored these variables and other potential covariates (length of hospital stay, admission to the NICU, and breastfeeding initiation) in the sensitivity analysis. This was restricted to children with data on these variables. An additional model was run, including those with missing data and coded as such. Due to the small sample size of the siblings’ cohort, the model was adjusted for variables with substantial Standardized Mean differences (SMD), including birthing parent age at birth, birthing parent mental disorders, sex and admission to the NICU. A list of the definitions of the variables is included in (Table 1 in S1 File).

### Statistical analysis

SMD was used to compare baseline demographic and clinical characteristics of the cohort (SMD > 0.1 was considered substantial) [23]. We used a 2-tailed *t t*est for continuous variables and categorical and dichotomous variables were described as proportions. Multivariable logistic regression models were used for all outcome models. For the analysis of sibling cohort data, a conditional logistic regression model was used to control for clustering of siblings within the same family. Multicollinearity was assessed using the variance inflation factor, and none met the criteria of >10.

### Secondary analysis

To better understand potential predictors of adverse developmental outcomes, we evaluated the association of the timing of opioid exposure, duration of exposure and cumulative opioid dose with developmental vulnerability. Firstly, we categorized children based on time of exposure into 3 groups: exposed during the first trimester, exposed during the second and/or third trimester and exposed throughout pregnancy. We estimated the last menstrual period (LMP) as the difference between birth date and gestational age. The first trimester included exposures between the LMP and 13 weeks of gestation, the second trimester included exposures between 14 and 27 weeks of gestation and the third trimester included exposures between 28 weeks of gestation and birth. Due to the limited number of exposures during the second and third trimester (5% and 7% of the exposure group, respectively), they were combined in the logistic regression model. Secondly, we dichotomized the duration of exposure into ‘acute’ < 30 days and ‘chronic’ ≥ 30days [24]. We further evaluated the association between cumulative MME and EDI outcomes. Exposed children were dichotomized by the median of the cumulative MME (<540 and ≥540) [25]. Finally, we calculated the E-value to examine the potential influence of unmeasured confounding [26].

A p-value < .05 was considered statistically significant. All statistical analyses were conducted using SAS, version 9.4 (SAS Institute Inc.).

## Results

### Description of study population

**Fig 1:**
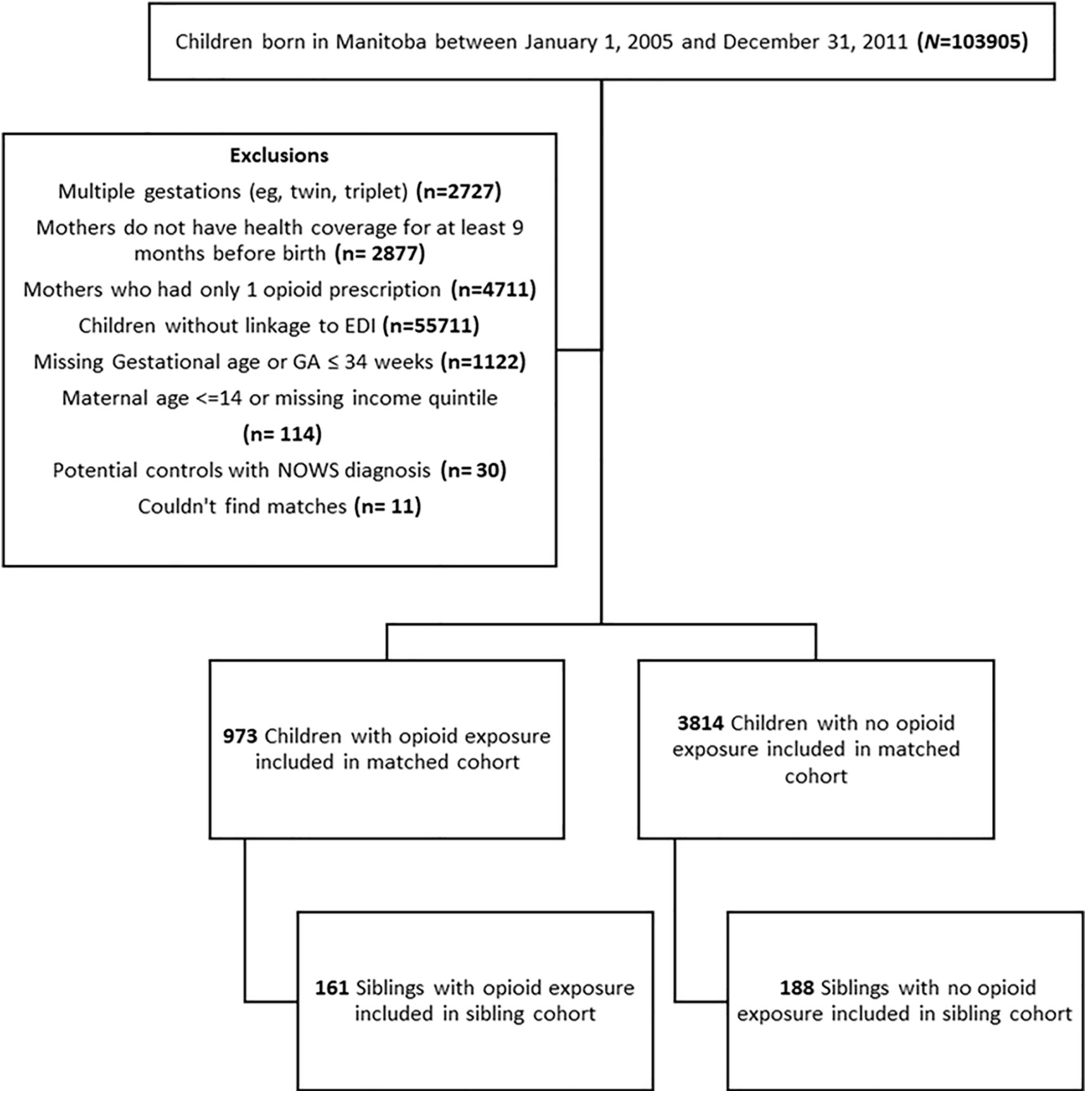
Flow diagram of the cohort.

Of the 103,905 eligible children born in Manitoba during the study period, 973 were included in the exposure group and 3,814 in the matched control group (Fig 1). The matching variables were balanced between the exposed and control groups (Table 1). PPOE comprised codeine (94%), oxycodone (8%), methadone (3%), morphine (2%), phenylpiperidine derivatives (1.5%), and hydromorphone (0.6%). Most exposures occurred during the first trimester (23%) or throughout pregnancy (31%). The lowest income quintile accounted for 48% of the exposure group dyads. Higher proportions of the birthing parents in the exposure group had diabetes (5% vs 3% of controls) and mental disorder diagnoses (34% vs 9% of controls). Birthing parent smoking and alcohol use during pregnancy was higher in the exposure group 38% and 17% compared with 18% and 13% in the control group, respectively. Receipt of child protection services was substantially higher in the exposure group; 57% of children with PPOE received child protection services, compared with 17% and of children in the control group. Children in the exposure group had longer hospital and NICU stays (SMD > 0.1). Breastfeeding initiation in hospital was lower in the exposure group 58% compared with 83% in the control group. Among children with PPOE, 6% had a NOWS diagnosis.

**Table 1:** Demographic and clinical characteristics of the matched cohort and sibling cohorts.

Characteristic	Matched cohort			Sibling cohort		
	Children with PPOE (*N* = 973)	Unexposed matched controls (*N* = 3814)	SMD	Children with PPOE (N = 161)	Unexposed siblings (N = 188)	SMD
Birthing parent						
Age at delivery, yr Median (IQR) Mean, (SD)	27 (23 –31) 27(5)	27 (23 –31) 27(5)	0.0	27 (24 –31) 27(5)	24 (19 –28) 24(5)	0.67
Age at delivery, yr N(%)						
≤19	47(5)	181(5)	NA	S	48(25)	NA
20-24	264(27)	1044(27)	NA	43(27)	56(30)	NA
25-29	309(32)	1213(32)	NA	56(34)	51(27)	NA
30-34	236(24)	927(24)	NA	40(24)	22(12)	NA
35-39	100(10)	387(10)	NA	14(8)	11(6)	NA
≥40	62(2)	17(2)	NA	S	S	NA
Geographical region						
Urban	634(65)	2489(65)	0.00	97(60)	106(56)	0.07
Income Quantile						
1(lowest)	466(48)	1825(48)	NA	58(36)	74(39)	NA
2	170(17)	666(17)	NA	33(20)	32(17)	NA
3	533(14)	136(14)	NA	27(17)	34(18)	NA
4	446(12)	113(12)	NA	23(14)	26(14)	NA
5(highest)	344(9)	88(9)	NA	20(12)	22(12)	NA
Exposure timing						
Exposure during the 1^ST^ Trimester	225(23)	__	NA	48(30)	__	NA
Exposure during the 2^nd^ Trimester	47(5)	__	NA	6(4)	__	NA
Exposure during the 3^rd^ Trimester	66(7)	__	NA	21(13)	__	NA
Exposure during 1^st^ and 2nd	177(18)	__	NA	28(17)	__	NA
Exposure during 1^st^ and 3rd	66(7)	__	NA	11(7)	__	NA
Exposure during 2nd and 3rd	95(10)	__	NA	25(15)	__	NA
Exposure throughout pregnancy	297(31)	__	NA	22(14)	__	NA
Cumulative MME Median (IQR) Mean, (SD)	540(270-1350) 2025(10817)	__ __	NA	369(270-792) 1030(2221)	__ __	NA
Type of opioid exposed to						
Morphine	21(2)	__	NA	6(3)	__	NA
Oxycodone	78(8)	__	NA	13(8)	__	NA
Phenylpiperidine derivatives	15(1.5)	__	NA	S	__	NA
Methadone	32(3)	__	NA	0	__	NA
Codeine	918(94)	__	NA	155(96)	__	NA
Hydromorphone	6(0.6)	__	NA	0	__	NA
Diabetes	52(5)	97(3)	0.14	6(3)	S	NA
Prenatal mental disorder diagnoses	329(34)	335(9)	0.66	27(17)	20(11)	0.18
Birthing parent Substance Use^a^	197(20)	152(4)	0.68	12(7)	11(6)	0.03
Alcohol use during pregnancy^b^	166(17)	489(13)	0.26	22 (14)	17(9)	0.03
Smoking^c^	366(38)	675(18)	0.77	54(33)	46(24)	0.03
Neonatal						
Gestational age Median (IQR) Mean, (SD)	39 (38 –40) 38.9(1.6)	39 (38 –40) 39.1(1.4)	0.14	39 (38 –40) 39(1.4)	40(39 –40) 39 (1.4)	0.0
MLP 35–36 weeks	76(8)	245(6)	NA	7(4)	8(4)	NA
Term 37–41	885(91)	3534(93)	NA	152(94)	178(95)	NA
42+	12(1)	35(1)	NA	S	S	NA
Sex Male	496(51)	1953(51)	0.00	75 (47)	91(48)	0.00
Birth Weight Median (IQR) Mean, (SD)	3416(3061-3748) 3416(538)	3463(3127-3798) 3472(520)	0.1	3446(3150-3792) 3497 (541)	3507(3220-3720) 3522(491)	0.05
Apgar score 1 Median (IQR) Mean, (SD)	9 (8 –9) 8(3)	9 (8 –9) 8(1)	0.01	9 (8 –9) 8(2)	9 (8 –9) 8(1)	0.07
Apgar score 5 Median (IQR) Mean (SD)	9 (9 –9) 9(3)	9 (9 –9) 9(2)	0.02	9 (9 –9) 9(1)	9 (9 –9) 9(1)	0.08
Receipt of child protection services *prior EDI assessment*	556(57)	640(17)	0.33	53(32)	64(34)	0.02
Length of hospital sta Median (IQR) Mean (SD)	2 (2 –3) 3.4(5)	2 (1 –3) 2.6(5.5)	0.15	2 (1 –3) 2.7(6)	2 (2 –3) 2.4(2.3)	0.06
Admission to NICU	161(17)	276(7)	0.29	15(9)	6(3)	0.25
NICU days Median (IQR) Mean (SD)	5 (2 –10) 8(10)	3 (1 –7) 6(8)	0.26	2 (1 –5) 8(19)	7 (3 –13) 8(8)	0.05
Breastfeeding initiation	559(58)	3141(83)	0.56	102(63)	125(66)	0.05
NOWS diagnosis	60(6)	__	NA	S	0(0)	NA

Abbreviations: IQR, Interquartile range; SD, Standard deviation; MME, Morphine milligram equivalents; NA, not applicable; S, suppressed data (for privacy reasons, data are suppressed for any count >1 but <5).

a Missing data for 51% subjects in the matched cohort and 78% in the sibling cohort.

b Missing data for 54% subjects in the matched cohort and 76% in the sibling cohort.

^c^Missing data for 50% subjects in the matched cohort and 74% in the sibling cohort.

### Logistic regression models

EDI outcomes revealed that 51% of children with PPOE were developmentally vulnerable in one or more domains compared to 32% of the control group (AOR 1.46; 95% CI 1.23-1.71) (Table 2). PPOE increased the risk of developmental vulnerability in all EDI domains, emotional maturity domain was the least affected (AOR,1.25;95% CI 1.01-1.55). Table 2 in S1 File. Presents the multivariable logistic regression model for developmental vulnerability in any 1 or more EDI domains. All EDI domain scores mean differences were significant for the matched cohort (p < .0001) (Table 3 in S1 File).

**Table 2:** EDI vulnerability results: Exposed versus matched control.

Vulnerability Outcomes	Exposed, n(%)	Control n(%)	Crude OR(95%CI)^a^	AOR(95%CI)^a,b^
Vulnerability in any EDI domain	497(51)	1223(32)	2.21(1.92-2.55)	1.46(1.23-1.71)
Vulnerability in 2 or more domains	313(32)	688(18)	2.16(1.84-2.52)	1.43 (1.12-1.60)
Vulnerability in the EDI Domain of				
Physical health and well-being	266(28)	578(15)	2.11(1.79-2.49)	1.31(1.08-1.59)
Communication skills and general knowledge	217(22)	509(13)	1.87(1.56-2.23)	1.53(1.25-1.88)
Emotional maturity	207(21)	489(13)	1.85(1.54-2.22)	1.25(1.01-1.55)
Language and cognitive development	252(26)	509(13)	2.29(1.93-2.71)	1.53(1.25-1.86)
Social competence	266(27)	526(14)	2.36(1.99-2.79)	1.60(1.32-1.95)

^a^matched on birthing parent age, gestational age, sex, kindergarten year, and income quintiles.

^b^adjusted for birthing parent mental disorders, birthing parent diabetes, gestational age, sex and children’s receipt of child protective services.

### Secondary analysis

The association between prescription prenatal opioid exposure (PPOE) and developmental vulnerability in one or more EDI domains was found to be significantly linked with exposure to opioids during both the first trimester (AOR 1.64;95% CI 1.23-2.19) and throughout pregnancy (AOR, 1.71;95% CI 1.30-2.24) (Table 3). Exposure during the second and third trimesters was significantly associated with vulnerability in the emotional maturity domain only. Compared with the unexposed group, the risk of developmental vulnerability increased depending on the duration of exposure (Table 4). Additionally, a gradient effect was observed for low vs high MME in all outcomes except physical health and well-being and emotional maturity domains (Table 5). Crude odds ratio for the effects of timing of exposure, duration of exposure, and cumulative opioid dose are provided in Table 4, Table 5, and Table 6 in S1 File respectively.

**Table 3:** EDI Vulnerability results: Effect of timing of opioid exposure.

Vulnerability Outcomes	1^st^ trimester n(%) (n = 225)	2^nd^ or 3^rd^ Trimester or both n(%) (n = 208)	throughout pregnancy n(%) (n = 297)	Control n(%) (n = 3814)	1^st^ trimester AOR(95%CI)	2^nd^ or 3^rd^ trimester AOR(95%CI)	throughout pregnancy AOR(95%CI)
Vulnerability in any EDI domain	119(52)	88(42)	169(57)	1223(32)	1.64(1.23-2.19)	1.14(0.84-1.53)	1.71(1.30-2.24)
Vulnerability in 2 or more domains	74(32)	51(25)	106(36)	688(18)	1.54(1.13-2.11)	1.08(0.76-1.52)	1.58(1.19-2.12)
Vulnerability in the EDI Domain of							
Physical health and well-being,	66(29)	38(18)	85(29)	578(15)	1.49(1.09-2.06)	0.86(0.59-1.26)	1.29(0.95-1.75)
Communication skills and general knowledge	61(27)	38(18)	66(22)	509(13)	2.01(1.45-2.78)	1.22(0.84-1.78)	1.56(1.13-2.17)
Emotional maturity	39(17)	45(22)	72(24)	489(13)	1.00(0.68-1.46)	1.47(1.03-2.12)	1.34(0.97-1.86)
Language and cognitive development	65(29)	36(18)	90(30)	509(13)	1.85(1.34-2.56)	1.01(0.68-1.48)	1.81(1.34-2.46)
Social competence	60(27)	48(23)	93(31)	526(14)	1.62(1.17-2.26)	1.43(1.01-2.03)	1.81(1.34-2.45)

Adjusted for birthing parent mental disorders, birthing parent diabetes, gestational age, sex and children’s receipt of child protective services.

**Table 4:** EDI Vulnerability results: Acute vs chronic exposure (<30 days vs 30 or more days).

Vulnerability Outcomes	Unexposed n(%) (n = 3814)	Acute exposure n(%) (n = 518)	Chronic exposure n(%) (n = 455)	Acute exposure AOR(95%CI)	Chronic exposure AOR (95%CI)
Vulnerability in any EDI domain	1223(32)	257(49)	240(52)	1.43(1.18-1.76)	1.48(1.18-1.85)
Vulnerability in 2 or more domains	688(18)	157 (30)	156(34)	1.36 (1.08-1.69)	1.55(1.14-1.75)
Vulnerability in the EDI Domain of					
Physical health and well-being	578(15)	134(26)	132(29)	1.36(1.09-1.69)	1.55(1.21-1.98)
Communication skills and general knowledge	509(13)	115(22)	102(22)	1.48(1.16-1.88)	1.61(1.22-2.12)
Emotional maturity	489(13)	106(20)	101(22)	1.23(0.96-1.59)	1.28(0.96-1.69)
Language and cognitive development	509(13)	132(26)	120(27)	1.52(1.21-1.94)	1.53(1.17-2.00)
Social competence	526(14)	127(25)	139(31)	1.43(1.12-1.82)	1.86(1.44-2.41)

Adjusted for birthing parent mental disorders, birthing parent diabetes, gestational age, sex and children’s receipt of child protective services.

**Table 5:** EDI Vulnerability results: Cumulative MME high vs low categories (72-540 and ≥540-24800).

Vulnerability Outcomes	Unexposed n(%) (n = 3814)	Low MME n(%) (n = 475)	High MME n(%) (n = 479)	Low MME AOR(95%CI)	High MME AOR (95%CI)
Vulnerability in any EDI domain	1230(32)	236(50)	254(53)	1.46(1.18-1.79)	1.52(1.22-1.89)
Vulnerability in 2 or more domains	693(18)	145(31)	163(34)	1.39(1.11-1.76)	1.53(1.20-1.94)
Vulnerability in the EDI Domain of					
Physical health and well-being	582(15)	129(27)	133(28)	1.36(1.08-1.73)	1.29(1.00-1.67)
Communication skills and general knowledge	512(13)	104(22)	110(23)	1.49(1.16-1.93)	1.63(1.24-2.13)
Emotional maturity	494(13)	95(20)	107(23)	1.22(0.93-1.58)	1.31(0.99-1.73)
Language and cognitive development	512(13)	117(25)	132(28)	1.48(1.16-1.89)	1.65(1.27-2.13)
Social competence	530(14)	120(26)	142(30)	1.51(1.18-1.93)	1.77(1.38-2.28)

Adjusted for birthing parent mental disorders, birthing parent diabetes, gestational age, sex and children’s receipt of child protective services.

### Sensitivity analysis

The association between PPOE and developmental vulnerability was retained in the sensitivity analysis based on a complete case analysis. The model adjusted for length of hospital stay, admission to the NICU, breastfeeding initiation, alcohol use, smoking, and substance use; in addition to the variables included in the main analysis (Table 7 in S1 File). Communication skills and general knowledge (AOR 1.36;95% CI 1.05-1.78), language and cognitive development (AOR 1.42;95% CI 1.09-1.84) and social competence domains (AOR 1.65;95% CI 1.29-2.12) were the most affected domains. However, there was no significant association between PPOE and emotional maturity domain (AOR 1.17;95% CI 0.88-1.54). Breastfeeding initiation at the hospital was associated with a reduced risk of developmental vulnerability (AOR 0.71;95% CI 0.59-0.87) (Table 8 in S1 File).

### Sibling cohort

Of the 973 children born with PPOE, 161 had siblings without documented exposure to prescription opioids during pregnancy (unexposed, n = 188). Siblings were born within 1–11 years. In the sibling cohort, the median (IQR) age at delivery 27 (24 –31) years for the exposed siblings vs 24 (19 –28) years for the unexposed siblings. Adjusted analyses revealed no differences between exposed and unexposed siblings; EDI outcomes revealed that 44% of children with PPOE were developmentally vulnerable in one or more domains compared to 48% of the unexposed siblings (AOR 0.92;95%CI 0.58-1.46) (Table 6). None of the EDI domain scores’ mean differences were statistically significant between the exposed and unexposed siblings (Table 9 in S1 File).

**Table 6:** EDI vulnerability results: Exposed versus unexposed siblings.

Vulnerability Outcomes	Children with PPOE (n = 161) n(%)	Unexposed siblings (n = 188) n(%)	Crude OR(95%CI)	AOR(95%CI)
Vulnerability in any EDI domain	71(44)	91(48)	0.82(0.54-1.26)	0.92(0.58-1.46)
Vulnerability in 2 or more domains	42(26)	54(28)	0.88(0.55-1.42)	0.91(0.54-1.52)
Vulnerability in the EDI domain				
Physical health and well-being	34(21)	48(25)	0.78(0.47-1.28)	0.81(0.48-1.39)
Communication skills and general knowledge	34(21)	39(21)	1.02(0.61-1.71)	0.94(0.53-1.65)
Emotional maturity	23(14)	31(16)	0.85(0.47-1.52)	0.80(0.42-1.51)
Language and cognitive development	38(24)	48(25)	0.92(0.56-1.49)	1.04(0.61-1.77)
Social competence	34(21)	43(23)	0.89(0.54-1.49)	0.93(0.53-1.62)

^b^adjusted for birthing parent age, birthing parent mental disorders, admission to NICU, and sex.

## Discussion

In this matched cohort study investigating teacher-reported kindergarten developmental outcomes among children exposed prenatally to prescription opioids, PPOE was associated with an increased risk of developmental vulnerability. PPOE‘s negative impact on developmental outcomes in this study has potential biological plausibility, given the significant association between PPOE during the first trimester and developmental vulnerability compared with exposures during later trimesters. While brain development occurs throughout pregnancy, the first trimester is a critical period for organogenesis and structural formation. Exposures in early pregnancy may disrupt these fundamental processes, potentially leading to a negative impact on long-term development [27]. This emphasizes the necessity for preconception counselling to mitigate the potential negative developmental impact. A gradient effect was observed with high MME and exposures to longer durations as well. The prevalence of NOWS in the cohort was low, which could be attributed to the exposure to low-potency opioids and the fact that some of the exposures occurred during the first trimester. NOWS develops in 5–20% of children exposed to prescription opioids [28].

The mechanisms underlying the impact of PPOE on long-term neurodevelopmental outcomes remain poorly understood. In animal models and human brain cell cultures in vitro, opioids have been shown to be associated with microglia and neuronal apoptosis [29–31], neurotransmitter dysregulation [32], decreased neurogenesis [33], altered myelination [34], and disrupted architecture of brain networks [31]. Furthermore, preclinical models have revealed impaired learning and memory in offspring exposed to opioids during pregnancy [35]. Opioid receptors are abundant in the brain, and it has been hypothesized that improper activation of opioid receptors can influence fetal brain development [36].

Our findings are consistent with Kang et al [37], who found that children exposed to prescription analgesic opioids are at risk of developing attention deficit hyperactivity disorder, autism spectrum disorder, intellectual disabilities and other neuropsychiatric disorders and the risk was mainly associated with high opioid dose, longer duration of exposure and opioid exposures during early pregnancy. No association between PPOE and developmental outcomes was observed in their sibling’s cohort. In our matched cohort most opioid exposures were analgesic opioids, a small number of children were exposed to OAT (3%), this precluded a stratified analysis to examine the impact of analgesic opioids versus OAT exposures. In a Norwegian population-based cohort study, of 64, 256 fifth-grade children, prescription analgesic prenatal opioid exposure, mainly codeine (90.5%), was associated with small reductions in literacy and numeracy scores in the first trimester and with chronic exposures [38]. These results align with our findings in a comparable cohort with mainly codeine exposures. In contrast, Wen et al [25] found no association between overall birthing parent analgesic opioid use and increased risk of neurodevelopmental disorders in early childhood. However, increased risk of neurodevelopmental disorders was observed in children exposed to opioids for longer duration and at higher doses.

To address unmeasured social, genetic, and other environmental factors, we explored this association using a sibling cohort design. In the sibling cohort, we did not observe differences in neurodevelopmental outcomes between exposed and unexposed siblings. This could be explained by unmeasured confounding. The E-value that measures *unmeasured confounding* was 1.71. This indicates that an unmeasured confounder associated with PPOE and neurodevelopmental outcomes with an odds ratio of 1.71 could theoretically shift the lower 95% CI to include the null value; however, a weaker confounder could not. It is important to note that the sibling cohort had a small sample size which limits the interpretation of our findings. These findings support the assumption that the increased risk of negative developmental impacts in cases of PPOE may not be due to opioid exposure alone. Several social determinants of health including socioeconomic status, caregiver education, housing and food security, parental mental health, and access to prenatal and pediatric healthcare services can all independently affect developmental outcomes [6,39,40]. Growing evidence indicates that children with PPOE are more likely to be raised in adverse social conditions, which can heighten the developmental risk beyond the direct biological effect of PPOE [6]. Therefore, our findings should be interpreted in light of both impact of PPOE and the boarder social context in which these children and their siblings are raised.

In our study design, we included a matched control group and the multivariable logistic regression model adjusted for potential confounders. Detangling social determinants of health from risks related to opioid use during pregnancy, however, remains challenging. The benefits of opioid use, particularly opioid agonist therapies, in reducing birthing parent mortality, preventing relapse and spread of infectious diseases, and optimizing pregnancy and infant outcomes [41] must be considered within these discussions alongside variables that are challenging to capture using administrative health data. For instance, genetic and epigenetic factors and other factors (i.e., parenting behaviour, birthing parent stress, and emotional dysregulation) [42,43] have been associated with childhood developmental outcomes; however, we could not control for such factors using administrative data.

Children with PPOE are often exposed to a plethora of risk factors that need to be approached through a family-centred harm reduction lens to mitigate the long-term negative sequelae of PPOE and concomitant confounders. Several interventions have been shown to ameliorate adverse developmental outcomes at the population level, such as home-visiting programs [44], food supplement programs in schools and low-income families [45], comprehensive/integrated care [46] and family-centered approaches [43]. Educational challenges may not be apparent until the children attend school. The effectiveness of support and intervention measures are less effective when applied later in the child’s academic journey [47]. Incorporating the life experiences, needs, and perceptions of these parents and children while creating interventions will promote the development of programs that are effective, practical, and tailored to their needs. Our study strongly underscores the need for an increase in capacity for early developmental screening by family physicians and pediatricians, and assessment and coordinated intervention by certified child & adolescent clinical health psychologists and developmental pediatricians for children with PPOE in Manitoba [48]. Additionally, it emphasises the need of an updated comprehensive Clinical Practice Guidelines for managing children with PPOE to enhance the overall health and well-being of families and children affected by opioid use during pregnancy [46].

### Strengths

This is the first Canadian study evaluating the long-term neurodevelopmental impact of PPOE using EDI data. We used a matched control study design and we adjusted for several confounders that have been associated with negative developmental outcomes in this population. The utilization of thoroughly validated and well-recognized international measures of educational performance enhances the generalizability of the results. The use of MCHP data enables long-term follow-up and minimizes attrition and detection bias while also enabling inclusion of various confounders.

### Limitations

The use of administrative data does not allow the capture of illicit or diverted opioid use or an in-depth assessment of the concomitant social determinants of health. We cannot guarantee that birthing parents who filled an opioid prescription took the medication, which may lead to exposure misclassification and an over-estimation of prenatal opioid exposure. Despite using a matched cohort and adjusting for covariates, we are unable to consider all unmeasured or residual confounding due to the unavailability of environmental and genetic factors in the data repository as well as high missingness in variables related to alcohol use, smoking and substance use during pregnancy. This limitation precluded adjusting for these variables in all models. The sibling cohort had a small sample size, and we only had data on prescription opioids. Unexposed siblings may have been exposed to non-prescription opioids. The EDI is administered throughout Manitoba’s public education system; students enrolled in private schools and some schools in Indigenous communities were not included. Furthermore, assessment of educational outcomes by teachers could potentially introduce bias. However, teachers were unaware of the exposure status. Therefore, any errors stemming from this bias would be random and non-differential. Finally, these findings may not be relevant to newer OAT medications as they were not captured in this dataset. Most exposures involved prescription codeine, these findings may not generalize to illicit opioids or opioid agonist therapy used for opioid use disorder.

## Conclusion

In this matched cohort study, PPOE was associated with an increased risk of developmental vulnerability. The impact of PPOE has been previously shown to not only related to the direct impact on the exposure of the brain to opioids, but also a combination of other biological, social, and environmental factors. Considering the simultaneous presence of these risk factors, it is of utmost importance to provide children with PPOE and their families early and long-term developmental screening and family-centered support to help ameliorate later educational challenges.

## Supporting information

S1 File Supplementary Content. **Table 1.** Demographic and Clinical Variables’ Definition. **Table 2.** Multivariable Logistic Regression Model Results for vulnerability in EDI in 1 or more domains: Exposed Versus Matched Control. **Table 3.** EDI Domain Scores for the Exposed and Matched Control Group. **Table 4.** EDI Vulnerability results: Effect of Timing of Opioid Exposure. **Table 5.** EDI Vulnerability results: Acute vs chronic exposure. **Table 6.** EDI Vulnerability results: Cumulative MME high vs low categories. **Table 7.** EDI Vulnerability results: Exposed Versus Matched Control (sensitivity analysis).**Table 8.** Multivariable Logistic Regression Model Results for vulnerability in EDI in 1 or more domains: Exposed Versus Matched Control – sensitivity analysis (complete case).**Table 9.** EDI Domain Scores for the Sibling Cohort.
(DOCX)

S1 Table STROBE statement—checklist of items that should be included in reports of cohort studies. (DOCX)

## Abbreviations

POE: Prenatal Opioid Exposure
EDI: Early Development Instrument
NOWS: Neonatal Opioid Withdrawal Syndrome
MME: Morphine Milligram Equivalents
MCHP: Manitoba Center for Health Policy
