# Supplementary material for: Teacher-reported kindergarten development among children exposed to prescription opioids during pregnancy
Source: PLoS One. 2026 Aug 6;21(8):e0355101. doi: 10.1371/journal.pone.0355101 (PMC13446689; doi:10.1371/journal.pone.0355101)
Supplement: Supplementary file 1 [file pone.0355101.s001.docx]

**Supplementary Content**

**eTable 1.** Demographic and Clinical Variables’ Definition

**eTable 2.** Multivariable Logistic Regression Model Results for vulnerability in EDI in 1 or more domains: Exposed Versus Matched Control

**eTable 3.** EDI Domain Scores for The Exposed and Matched Control Group

**eTable 4.** EDI Vulnerability results: Effect of Timing of Opioid Exposure

**eTable 5.** EDI Vulnerability results: Acute vs Chronic Exposure (<30 days vs 30 or more days)

**eTable 6.** EDI Vulnerability results: Cumulative MME High vs Low Categories (72-540 and ≥540-24800)

**eTable 7.** EDI Vulnerability results: Exposed Versus Matched Control (sensitivity analysis)

**eTable 8.** Multivariable Logistic Regression Model Results for vulnerability in EDI in 1 or More Domains: Exposed Versus Matched Control -sensitivity analysis (complete case)

**eTable 9.** EDI Domain Scores for The Sibling Cohort

**eTable 10.** Multivariable Logistic Regression Model Results for vulnerability in EDI in 1 or more domains: Exposed Versus Unexposed Siblings

**eTable 11.** Opioid ATC codes

**eTable 1.** Demographic and Clinical Variables’ Definition and Databases Used in the Study

| Variable | Definition |
| --- | --- |
| Maternal age at delivery (years) | A record in Hospital Discharge Abstract Database |
| Geographical region (Urban or Rural) | As recorded in Manitoba Health Insurance Registry |
| Birthing parent prenatal mental health (yes/no)^1,2^ | During the gestational period:   - one or more hospitalizations with a diagnosis for depressive disorder, affective psychoses, neurotic depression, or adjustment reaction (ICD-9-CM codes 296.2-296.8, 300.4, 309, 311; ICD-10-CA codes F31, F32, F33, F34.1, F38.0, F38.1, F41.2, F43.1, F43.2, F43.8, F53.0, F93.0); OR - one or more physician visits with a diagnosis for depressive disorder, affective psychoses, or adjustment reaction (ICD-9-CM codes 296, 309 or 311); OR - one or more hospitalizations with a diagnosis for anxiety disorders (ICD-9-CM code 300; ICD-10-CA codes F32.0, F34.1, F40, F41, F42, F44, F45.0, F451, F452, F48, F68.0, F99); OR - one or more prescriptions for an antidepressant or mood stabilizer (ATC codes N03AB02, N03AB52, N03AF01, N05AN01, N06A) OR - one or more physician visits with a diagnosis for anxiety disorders (ICD-9-CM code 300) and one or more prescriptions for an antidepressant or mood stabilizer (ATC codes N03AB02, N03AB52, N03AF01, N05AN01, N06A); OR - one or more hospitalizations with a diagnosis for anxiety states, phobic disorders, or obsessive-compulsive disorders (ICD-9-CM codes 300.0, 300.2, 300.3; ICD-10-CA codes F40, F41.0, F41.1, F41.3, F41.8, F41.9, F42); OR - two or more physician visits with a diagnosis for anxiety disorders (ICD-9-CM code 300). |
| Birthing parent diabetes^3^ (yes/no) | Combination of any pre-gestational diabetes: in the time period 2 years prior to 25 weeks’ gestation:   - Had 1 or more hospitalization with a diagnosis of ICD–9–CM code 250; ICD–10 CA codes E10–E14; OR - Had 2 or more physician visits with a diagnosis of ICD–9–CM code 250; OR - Had 1 or more prescriptions for drugs used in diabetes, e.g., insulin, blood glucose lowering drugs (ATC code A10 excluding metformin), OR - Had 1 or more prescriptions for metformin with at least one physician visit for diabetes, OR - Had 1 or more glycohemoglobin (HbA1c) tests with a result ≥ 6.5%, OR - any gestational diabetes, meets the above definition from 26 weeks gestation to delivery AND did not meet the pre-gestational diabetes definition. |
| Income quintile (IQ)  (Categorical 1-5)^4^ at delivery | It is a measure of neighbourhood socioeconomic status that divides the population into 5 income groups (from lowest income to highest income) so that approximately 20% of the population is in each group.  Four data sources are required to generate IQ:   - the Manitoba Health Insurance Registry Data, - the public-use [Canada Census data](http://mchp-appserv.cpe.umanitoba.ca/viewDefinition.php?definitionID=103915) files developed by Statistics Canada (used to identify the average household income value by Dissemination Area (DA)) - the [Postal Code Conversion File (PCCF)](http://mchp-appserv.cpe.umanitoba.ca/viewDefinition.php?definitionID=104540) developed by Statistics Canada is used to identify and verify postal codes that fit within Dissemination Areas (DA) - the [Manitoba Health Population File](http://mchp-appserv.cpe.umanitoba.ca/viewDefinition.php?definitionID=104756) is used to identify the population of Manitoba at the time the income quintiles are generated.   The data are coded into one of the following groups:  Urban Income Quintiles: U1 (lowest) to U5 (highest);  Rural Income Quintiles: R1 (lowest) to R5 (highest); or  Income Not Found (NF). |
| Smoking during pregnancy  Categorical (yes/no) | As recorded in Familied First Screen Database |
| Alcohol use during pregnancy  Categorical (yes/no) |  |
| Birthing parent substance use ^5^  (yes/no) | - ‘illicit drug use during pregnancy’ recorded on Families First Screen OR - one or more hospitalizations during pregnancy with a diagnosis for drug psychoses, drug dependence, or nondependent abuse of drugs, using: - ICD-9-CM codes: 292 (drug psychoses), 304 (drug dependence), or 305 (nondependent abuse of drugs); OR - ICD-10-CA codes: F10-19 (mental and behavioral disorders due to psychoactive substance use) and F55 (abuse of non-dependence-producing substances) and Z50.3 (drug rehabilitation); OR - One or more physician visits during pregnancy with a diagnosis for drug psychoses, or drug dependence, or nondependent abuse of drugs, using ICD-9-CM codes 292, 304 or 305 |
| Care by Child and Family Services  Categorical (yes/no) | Receipt of child protection services as recorded in the Child and Family Services Database |
| Neonatal variables | |
| Sex | As recorded in Hospital Discharge Abstract Database |
| Gestational age |  |
| Birth weight |  |
| Apgar score 1 min and 5 min |  |
| Length of hospital stay |  |
| Admission to NICU |  |
| Length of NICU stay |  |
| Breastfeeding initiation |  |
| NAS diagnosis |  |

**eTable 2.** Multivariable Logistic Regression Model Results for vulnerability in EDI in 1 or more domains: Exposed Versus Matched Control

| Variables | Adjusted OR | 95% CI | P-value |
| --- | --- | --- | --- |
| Prenatal opioid exposure | 1.46 | 1.23-1.71 | 0.0001 |
| Gestational age | MLP (35-36) Ref  Term ≥37 1.01 | 0.79-1.30 | 0.95 |
| Male Sex | 1.97 | 1.74-2.23 | <.0001 |
| Maternal diabetes | 1.95 | 1.37-2.78 | 0.0002 |
| Maternal mental health | 1.09 | 0.90-1.30 | 0.40 |
| Receipt of CFS | 2.90 | 2.50-3.36 | <.0001 |

**eTable 3.** EDI Domain Scores for The Exposed and Matched Control Group

|  | Exposure group  (N=973) | Unexposed Matched control group  (N=3814) | Mean difference  (95% CI) | P-value |
| --- | --- | --- | --- | --- |
| EDI domains | | | | |
| Physical health and well-being, mean(SD) | (n= 971)  7.8 (1.8) | (n= 3811)  8.5(1.5) | 0.76(0.64-0.89) | <.0001 |
| Communication skills and general knowledge, mean(SD) | (n= 971)  6.3(2.9) | (n= 3811)  7.4(2.7) | 1.16(0.95-1.36) | <.0001 |
| Emotional maturity, mean(SD) | (n= 965)  7.2(1.8) | (n= 3802)  7.8(1.6) | 0.58(0.46-0.71) | <.0001 |
| Language and cognitive development, mean(SD) | (n=965)  7.1(2.6) | (n=3800)  8.02(2.1) | 0.95(0.77-1.13) | <.0001 |
| Social competence, mean(SD) | (n=969)  7.1(2.3) | (n=3810)  8.2(1.9) | 1.03(0.87-1.19) | <.0001 |

**eTable 4.** EDI Vulnerability results: Effect of Timing of Opioid Exposure Crude Odds Ratio

| Vulnerability Outcomes | 1^st^ trimester n(%)  (n=225) | 2^nd^ ,3^rd^  Trimester  or both  n(%)  (n=208) | throughout pregnancy  n(%)  (n=297) | Control n(%)  (n=3814) | 1^st^ trimester Crude OR(95%CI) ^a^ | 2^nd^ or 3^rd^  trimester  Crude OR(95%CI) ^a^ | throughout pregnancy  Crude OR(95%CI) ^a^ |
| --- | --- | --- | --- | --- | --- | --- | --- |
| Vulnerability in any EDI domain | 119(52) | 88(42) | 169(57) | 1223(32) | 2.38(1.82-3.12) | 1.55(1.17-2.06) | 2.79(2.20-3.55) |
| Vulnerability in 2 or more domains | 74(32) | 51(25) | 106(36) | 688(18) | 2.23(1.67-2.97) | 1.47(1.06-2.04) | 2.52(1.96-3.24) |
| Vulnerability in the  EDI Domain of | | | | | | | |
| physical health and well-being, | 66(29) | 38(18) | 85(29) | 578(15) | 2.32(1.72-3.13) | 1.25(0.87-1.79) | 2.25(1.72-2.94) |
| communication skills and general knowledge | 61(27) | 38(18) | 66(22) | 509(13) | 2.43(1.78-3.31) | 1.46(1.01-2.09) | 1.85(1.38-2.48) |
| emotional maturity | 39(17) | 45(22) | 72(24) | 489(13) | 1.44(1.00-1.05) | 1.92(1.36-2.71) | 2.17(1.63-2.87) |
| language and cognitive development | 65(29) | 36(18) | 90(30) | 509(13) | 2.64(1.95-3.58) | 1.39(0.95-2.01) | 2.81(2.16-3.66) |
| social competence | 60(27) | 48(23) | 93(31) | 526(14) | 2.29(1.68-3.12) | 1.89(1.37-2.65) | 2.85(2.19-3.69) |
| ^a^ matched on birthing parent age, gestational age, sex, kindergarten year, and income quintiles. | | | | | | | |

**eTable 5.** EDI Vulnerability results: Acute vs Chronic Exposure (<30 days vs 30 or more days)

| Vulnerability Outcomes | Unexposed  n(%)  (n=3814) | Acute exposure n(%)  (n=518) | Chronic  exposure  n(%)  (n=455) | Acute exposure Crude OR(95%CI) ^a^ | Chronic exposure Crude OR (95%CI) ^a^ |
| --- | --- | --- | --- | --- | --- |
| Vulnerability in any EDI domain | 1223(32) | 257(49) | 240(52) | 2.09(1.74-2.52) | 2.35(1.93-2.86) |
| Vulnerability in 2 or more domains | 688(18) | 157 (30) | 156(34) | 1.98(1.61-2.43) | 2.35(1.91-2.91) |
| Vulnerability in the  EDI Domain of | | | | | |
| physical health and well-being | 578(15) | 134(26) | 132(29) | 1.95(1.57-2.43) | 2.29(1.84-2.86) |
| communication skills and general knowledge | 509(13) | 115(22) | 102(22) | 1.85(1.47-2.33) | 1.87(1.47-2.37) |
| emotional maturity | 489(13) | 106(20) | 101(22) | 1.75(1.38-2.21) | 1.97(1.54-2.50) |
| language and cognitive development | 509(13) | 132(26) | 120(27) | 2.22(1.79-2.77) | 2.33(1.85-2.94) |
| social competence | 526(14) | 127(25) | 139(31) | 2.03(1.63-2.53) | 2.76(2.21-3.44) |
| ^a^ matched on birthing parent age, gestational age, sex, kindergarten year, and income quintiles. | | | | | |

**eTable 6.** EDI Vulnerability results: Cumulative MME High vs Low Categories (72-540 and ≥540-24800)

| Vulnerability Outcomes | Unexposed  n(%)  (n=3814) | Low MME n(%)  (n=475) | High MME  n(%)  (n=479) | Low MME Crude OR(95%CI) ^a^ | High MME Crude OR(95%CI) ^a^ |
| --- | --- | --- | --- | --- | --- |
| Vulnerability in any EDI domain | 1230(32) | 236(50) | 254(53) | 2.09(1.72-2.53) | 2.39(1.97-2.89) |
| Vulnerability in 2 or more domains | 693(18) | 145(31) | 163(34) | 1.99(1.62-2.46) | 2.34(1.90-2.87) |
| Vulnerability in the  EDI Domain of | | | | | |
| physical health and well-being | 582(15) | 129(27) | 133(28) | 2.08(1.67-2.59) | 2.16(1.73-2.69) |
| communication skills and general knowledge | 512(13) | 104(22) | 110(23) | 1.82(1.43-2.30) | 1.94(1.54-2.45) |
| emotional maturity | 494(13) | 95(20) | 107(23) | 1.69(1.33-2.17) | 1.98(1.57-2.51) |
| language and cognitive development | 512(13) | 117(25) | 132(28) | 2.12(1.68-2.66) | 2.51(2.01-3.13) |
| social competence | 530(14) | 120(26) | 142(30) | 2.11(1.68-2.64) | 2.66(2.14-3.30) |
| ^a^ matched on birthing parent age, gestational age, sex, kindergarten year, and income quintiles. | | | | | |

**eTable 7.** EDI Vulnerability results: Exposed Versus Matched Control (sensitivity analysis)

| Vulnerability Outcomes | Exposed, n(%)  (n=585) | Control n(%)  (n=3004) | AOR(95%CI)^a^ | AOR(95%CI)^b^ |
| --- | --- | --- | --- | --- |
| Vulnerability in any EDI domain | 290(50) | 880(30) | 1.37(1.11-1.70) | 1.28(1.08-1.52) |
| Vulnerability in 2 or more domains | 184(31) | 493(16) | 1.34 (1.05-1.69) | 1.27 (1.05-1.53) |
| Vulnerability in the  EDI Domain of | | | | |
| physical health and well-being | 266(28) | 578(15) | 1.32(1.03-1.71) | 1.22(1.00-1.49) |
| communication skills and general knowledge | 125(21) | 377(13) | 1.36(1.05-1.78) | 1.37(1.11-1.69) |
| emotional maturity | 121(21) | 349(12) | 1.17(0.88-1.54) | 1.09(0.87-1.36) |
| language and cognitive development | 144(24) | 362(12) | 1.42(1.09-1.84) | 1.37(1.12-1.68) |
| social competence | 163(28) | 365(12) | 1.65(1.29-2.12) | 1.42(1.16-1.74) |
| ^a^ Complete case analysis  ^b^ Coded as missing  ^a,b^ matched on birthing parent age, gestational age, sex, kindergarten year, and income quintiles.  ^a,b^ adjusted for birthing parent mental disorders, birthing parent diabetes, gestational age, sex and children’s receipt of child protective services, length of hospital stay, admission to the NICU, breastfeeding initiation, alcohol use, smoking, and substance use. | | | | |

**eTable 8.** Multivariable Logistic Regression Model Results for vulnerability in EDI in 1 or more domains: Exposed Versus Matched Control -sensitivity analysis (complete case)

|  | Adjusted OR | 95% CI | P-value |
| --- | --- | --- | --- |
| Prenatal opioid exposure | 1.37 | 1.11-1.70 | 0.004 |
| Gestational age | MLP (35-36) Ref  Term ≥37 1.18 | 0.85-1.64 | 0.31 |
| Male Sex | 2.08 | 1.78-2.42 | <.0001 |
| Maternal diabetes | 2.06 | 1.28-3.30 | 0.003 |
| Maternal mental health | 1.16 | 0.93-1.46 | 0.19 |
| Maternal substance use | 1.07 | 0.78-1.44 | 0.68 |
| Alcohol use | 1.01 | 0.83-1.23 | 0.94 |
| Smoking | 1.39 | 1.16-1.68 | 0.0004 |
| Receipt of CFS | 2.77 | 2.26-3.41 | <.0001 |
| NICU admission | 0.73 | 0.53-1.01 | 0.06 |
| Length of hospital stay | 1.01 | 0.99-1.04 | 0.28 |
| Breastfeeding initiation | 0.71 | 0.59-0.87 | 0.0009 |

**eTable 9.** EDI Domain Scores for The Sibling Cohort

|  | Children with POE  (N=161) | Unexposed siblings  (N=188) | Mean difference  (95% CI) | P-value |
| --- | --- | --- | --- | --- |
| Physical health and well-being, mean(SD) | 8.0(1.9) | 8.1(1.8) | 0.13(-0.25-0.51) | 0.5 |
| Communication skills and general knowledge, mean(SD) | 6.7(2.9) | 6.5(3.1) | -0.14(-0.78-0.49) | 0.6 |
| Emotional maturity, mean(SD) | 7.6(1.7) | 7.6(1.7) | 0.04(-0.3-0.4) | 0.8 |
| Language and cognitive development, mean(SD) | 7.3(2.6) | 7.2(2.5) | -0.12(-0.6-0.41) | 0.6 |
| Social competence, mean(SD) | 7.5(2.3) | 7.5(2.3) | 0.01(-0.46-0.48) | 0.9 |

**eTable 10.** Multivariable Logistic Regression Model Results for vulnerability in EDI in 1 or more domains: Exposed Versus Unexposed Siblings

| Variables | Adjusted OR | 95% CI | P-value |
| --- | --- | --- | --- |
| Prenatal opioid exposure | 0.92 | 0.58-1.46 | 0.74 |
| Maternal age at birth | 0.96 | 0.93-1.01 | 0.08 |
| Male Sex | 1.91 | 1.24-2.93 | 0.004 |
| Admission to NICU | 1.08 | 0.44-2.65 | 0.87 |
| Maternal mental health | 1.02 | 0.54-1.91 | 0.95 |

**eTable 11.** Opioid ATC codes

| Opioids prescribed during pregnancy | ATC Code |
| --- | --- |
| Methadone | Methadone N07BC02  Levomethadone N07BC05  Methadone combinations excl. psycholeptics N02AC52  Normethadone R05DA06 |
| Buprenorphine | Buprenorphine N02AE01 (analgesic opioid)  Buprenorphine N07BC01 (used in opioid dependence)  Buprenorphine, combinations N07BC51 |
| Codeine | Acetyldihydrocodeine R05DA12  Codeine R05DA04  Codeine and acetylsalicylic acid N02AJ07  Codeine and ibuprofen N02AJ08  Codeine and other non-opioid analgesics N02AJ09  Codeine and paracetamol N02AJ06  Codeine, combinations excl. psycholeptics N02AA59  Codeine , combinations with psycholeptics N02AA79  Dihydrocodeine N02AA08  Dihydrocodeine and acetylsalicylic acid N02AJ02  Dihydrocodeine and other non-opioid analgesics N02AJ03  Dihydrocodeine and paracetamol N02AJ01  Dihydrocodeine, combinations N02AA58 |
| Hydrocodone | R05DA03 |
| Hydromorphone | Hydromorphone N02AA03  Hydromorphone and antispasmodics N02AG04  Hydromorphone and naloxone N02AA53 |
| Morphine | Morphine N02AA01  Morphine and antispasmodics N02AG01  Morphine, combinations A07DA52 (as antipropulsive)  Morphine, combinations N02AA51(analgesics)  Nicomorphine N02AA04  Apomorphine N04BC07 (as dopamine agonist)  Diamorphine N07BC06  Ethylmorphine R05DA01  Opium alkaloids with morphine R05DA05 |
| Oxycodone | Oxycodone N02AA05  Oxycodone and acetylsalicylic acid N02AJ18  Oxycodone and Ibuprofen N02AJ19  Oxycodone and naloxone N02AA55  Oxycodone and naltrexone N02AA56  Oxycodone and paracetamol N02AJ17 |
| Pentazocine | N02AD01 |
| Dextropropoxyphene | Dextropropoxyphene N02AC04  Dextropropoxyphene, combinations excl. psycholeptics N02AC54  Dextropropoxyphene, combinations with psycholeptics N02AC74 |
| Tapentadol | N02AX06 |
| Phenylpiperidine derivatives | Pethidine N02AB02  Ketobemidone N02AB01  Fentanyl N02AB03  Pethidine, combinations excl. psycholeptics N02AB52  Pethidine, combinations with psycholeptics N02AB72 |

**References**

1.	Term: Prenatal Psychological Distress. Accessed March 23, 2024. http://mchp-appserv.cpe.umanitoba.ca/viewDefinition.php?definitionID=104497

2.	perinatal_report_WEB.pdf. Accessed March 23, 2024. http://mchp-appserv.cpe.umanitoba.ca/reference/perinatal_report_WEB.pdf

3.	Dart AB, Ruth CA, Sellers EA, Au W, Dean HJ. Maternal diabetes mellitus and congenital anomalies of the kidney and urinary tract (CAKUT) in the child. *Am J Kidney Dis*. 2015;65(5):684-691. doi:10.1053/j.ajkd.2014.11.017

4.	Concept: Income Quintiles - Child Health Income Quintiles. Accessed March 23, 2024. http://mchp-appserv.cpe.umanitoba.ca/viewConcept.php?printer=Y&conceptID=1161

5.	Concept: Substance Use Disorders / Substance Abuse - Measuring Prevalence. Accessed March 23, 2024. http://mchp-appserv.cpe.umanitoba.ca/viewConcept.php?conceptID=1471
